# A symmetric direct ammonia fuel cell enabled with poisoning-tolerant PtCo/C electrodes

**DOI:** 10.1039/d6ta04565k

**Published:** 2026-08-04

**Authors:** Huimin Zhang, Peimiao Zou, Yingjie Song, Yisong Han, Marc Walker, Shanwen Tao

**Affiliations:** a School of Engineering, University of Warwick Coventry CV4 7AL UK S.Tao.1@warwick.ac.uk; b School of Civil Engineering and Architecture, East China Jiao Tong University Nanchang Jiangxi 330013 China; c Electron Microscopy Research Technology Platform, Department of Physics, University of Warwick Coventry UK; d Photoemission Research Technology Platform, Department of Physics, University of Warwick Coventry UK; e Hartnoll Centre, University of Warwick Coventry CV4 7AL UK

## Abstract

The ammonia oxidation reaction (AOR) is a key anodic process in low-temperature direct ammonia fuel cells (DAFCs), and its catalytic efficiency directly determines the energy conversion performance. Although a well-established structure–activity relationship exists between Pt-based materials and AOR behavior, pure Pt still suffers from *N intermediate poisoning and high precious metal loading. Herein, we report a nanostructured PtCo solid-solution catalyst synthesized *via* a two-step pyrolysis strategy. Structural characterization studies reveal that the incorporation of Co atoms into the Pt face-centered cubic lattice induces lattice contraction and a negative shift in the Pt 4f binding energy, which weakens the Pt–N bond strength. The optimized PtCo/C-3 catalyst exhibits a low onset potential of 0.50 V *vs.* RHE and a peak current density of 79.3 mA cm^−2^ for alkaline AOR, while retaining approximately 90% of its initial activity after 400 cycles. In a DAFC test, a symmetric cell employing the PtCo/C-3 catalyst as both the anode and cathode delivers an open-circuit voltage of 0.56 V and a maximum power density of 47.9 mW cm^−2^ at 90 °C. This work reveals a viable route to modulate the electronic structure of Pt *via* trace 3d-metal alloying, simultaneously mitigating poisoning and enhancing intrinsic AOR activity, offering a new paradigm for poisoning-tolerant anode design for DAFCs.

## Abstract 2

A poisoning-tolerant PtCo/C catalyst synthesized *via* a two-step pyrolysis method achieves high mass activity for ammonia oxidation, and is used as electrodes for a symmetric direct ammonia fuel cell with excellent performance.

## Graphical abstract

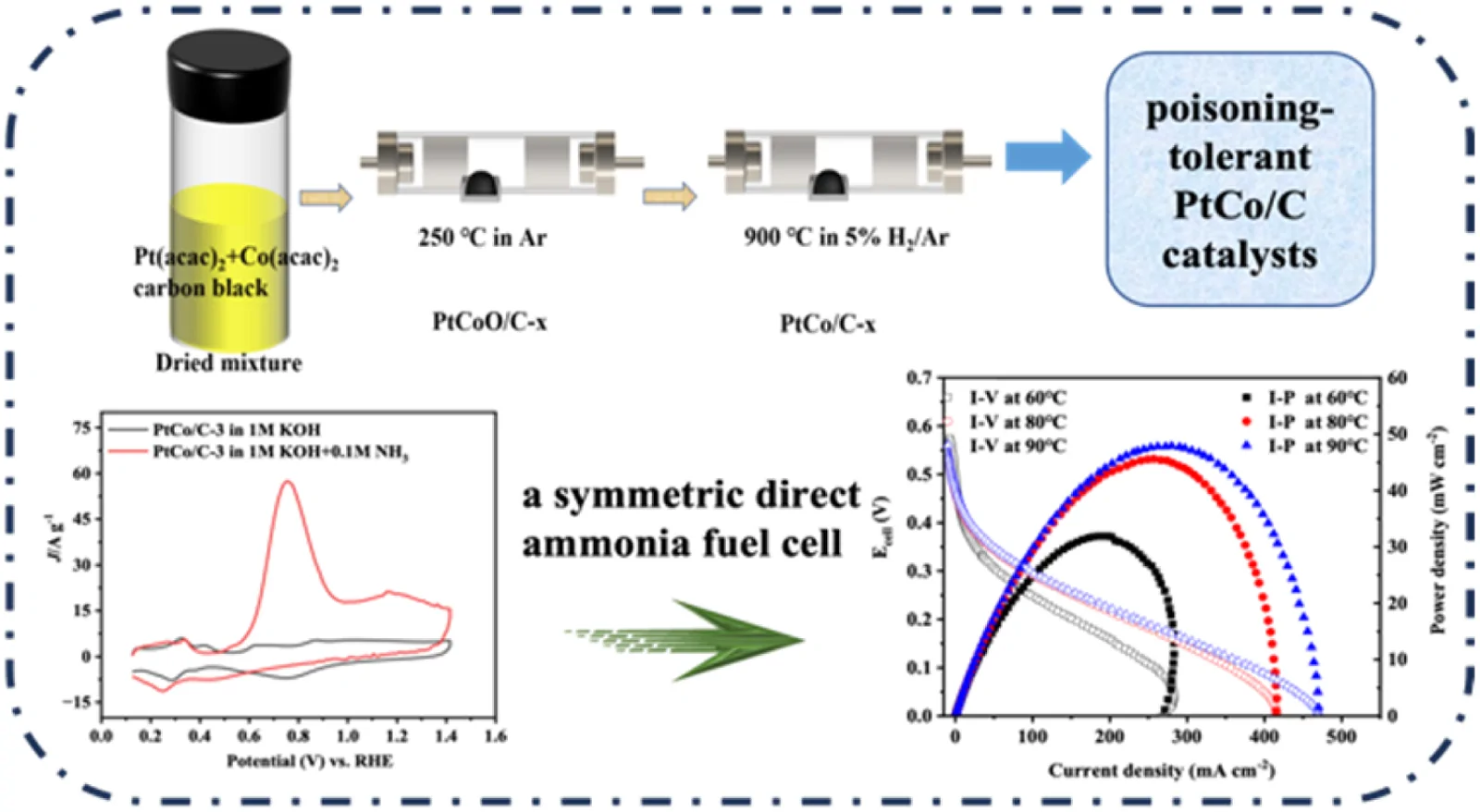

## 1 Introduction

Ammonia (NH_3_), as a carbon-free hydrogen-rich compound, offers advantages such as high hydrogen content, ease of liquefaction for storage and transport, and relatively mature infrastructure, making it an important carrier for linking renewable energy to hydrogen utilization.^1–5^ However, achieving the efficient and direct conversion of the chemical energy stored in ammonia into electrical energy remains challenging. Consequently, ammonia fuel-cell technology has attracted considerable attention in recent years. In low-temperature direct ammonia fuel cells (DAFCs), ammonia can be directly supplied as an anode fuel,^6–9^ eliminating the need for an external cracking unit to produce hydrogen. This configuration simplifies the overall system and holds great promise for applications in distributed power generation and high-safety energy storage.^2,3,8–11^ However, further development of DAFCs remains limited by the intrinsically sluggish and complex kinetics of the anodic ammonia oxidation reaction (AOR).^8,12,13^ The AOR involves multi-step dehydrogenation, electron transfer, and N–N coupling processes. Its reaction pathway is highly dependent on the electronic structure of the catalyst surface and the adsorption behavior of intermediates, which can lead to different product distributions, such as N_2_ and NO_2_^−^/NO_3_^−^.^14–17^ Previous studies on Pt-based alloy and interfacial catalysts have suggested that lattice modification, metal–metal electronic interaction, and interface effects can regulate the adsorption strength of nitrogen-containing intermediates and improve the poisoning tolerance of AOR catalysts.^18,19^ For ammonia oxidation, excessively strong interaction with adsorbed species such as *NH_*x*_ and *N may block active sites and retard subsequent reaction steps. For an efficient AOR catalyst, sufficient N–H bond activation capability is required. However, excessively strong adsorption of deeply dehydrogenated intermediates, especially *N species, must be avoided; otherwise, surface poisoning, aggravated anodic polarization, and decay in device output may occur.^20,21^ Therefore, achieving a balance between “promoting ammonia activation” and “mitigating intermediate poisoning” is the core scientific issue in AOR catalyst design.^22,23^ Among existing low-temperature AOR catalysts, Pt-based materials remain the most representative, primarily due to Pt's high activity for N–H bond cleavage and the well-established structure–activity relationship between its surface structure and AOR behavior.^22,24–27^ Studies have shown that the ammonia oxidation activity and intermediate coverage behavior of Pt-based catalysts depend strongly on the crystal facet, size, and electronic state of Pt nanocrystals. Moreover, pronounced structural sensitivity has also been observed for Pt/C nanoparticles with specific morphologies.^15,28,29^ However, monometallic Pt is limited by high precious metal loading, severe *N poisoning, and poor selectivity. Modulating the electronic structure and surface adsorption behavior of Pt by alloying with a second metal therefore offers a promising strategy to enhance AOR performance.^14,30^

Alloying, particularly with 3d transition metals (Pt–M, where M = Ni,^31–33^ Co,^22,34,35^ Cu,^36^ Zn,^34^*etc.*), is considered an effective approach to optimize Pt-based AOR catalysts. Cobalt, owing to its strong electronic modulation capability and relatively low cost, has received sustained attention in recent years. Specifically, the introduction of Co into PtCo alloys can modify the surface electronic state of Pt, weaken the Pt–N bond strength, and alleviate the excessively strong adsorption of deeply dehydrogenated species. Additionally, the surface oxidation state of Co may participate in surface dehydrogenation and site regeneration, further enhancing poisoning resistance.^22,35,37,38^ Recent studies have shown that the PtCo system can undergo a temperature-induced morphological transformation from core–shell to hollow structures, significantly increasing the electrochemically active surface area and DAFC output.^37^ In parallel, the upshifted d-band edge of PtCo alloys has been identified as an effective electronic structural descriptor for AOR activity, confirming that Co introduction not only alters the geometric structure but also intrinsically modifies the reaction kinetics.^22^ Moreover, emerging strategies—including constructing PtCo–CoO_*x*_ and PtCo–C dual interfaces and employing trace Co substitution to form Pt alloys—have further highlighted the unique role of Co in promoting AOR and boosting DAFC power density.^35,38^

Although PtCo catalysts exhibit clear advantages in the AOR and DAFCs, several critical gaps remain in current research. First, the synergistic effects of Pt/Co atomic ratios, the degree of alloying, and surface Co chemical states on AOR activity, poisoning resistance, and product selectivity have yet to be fully elucidated.^14,22,37,38^ Second, the adsorption–conversion behavior of key intermediates on PtCo nanocatalysts and its correlation with the electronic structure has not been fully elucidated.^14,22,38^ More importantly, most studies remain at the half-cell level, yet how these structural advantages translate into high power output and long-term stability at the DAFC device level requires more in-depth structure–performance relationship studies.^16,21,35,37^ Additionally, most existing PtCo catalysts are fabricated *via* conventional impregnation or one-step co-reduction methods, which often lead to uneven alloy particle distribution and uncontrollable metal alloying degree. Differently, we adopt a modified solvothermal route with precise metal precursor ratio regulation to synthesize uniform PtCo alloy nanoparticles with optimized electronic modulation, which simultaneously balances AOR and ORR intrinsic activity. In addition, the majority of published PtCo-related DAFC research only adopts asymmetric electrode configurations with separate anode and cathode catalysts, while this work systematically assembles a fully symmetric DAFC using the as-prepared single PtCo/C bifunctional catalyst on both sides. Supported by variable-temperature cell performance and impedance data, we comprehensively analyze the trade-off between temperature-promoted electrode kinetics and increasing FAA membrane resistance, as well as the poisoning effect of ammonia crossover on the cathodic ORR in a symmetric MEA.

## 2 Experimental

### 2.1 PtCo/C catalyst synthesis and physical characterization

#### 2.1.1 PtCo/C catalyst synthesis

In a typical synthesis procedure, 80 mg of Pt(acac)_2_ and 27 mg of Co(acac)_2_ were first dissolved in 15 mL of acetone *via* ultrasonication for 5 minutes. Subsequently, 50 mg of the Vulcan XC 72R carbon support was added, and the mixture was ultrasonicated for an additional 10 minutes to ensure homogeneous dispersion.

The resulting suspension was then transferred to a hotplate and heated at 55 °C to evaporate the acetone. After complete evaporation of the solvent, the solid residue, containing the carbon support and metal precursors, was collected and thoroughly ground with an agate mortar. This obtained power was placed in a quartz tube and annealed under a flowing argon atmosphere. The temperature was ramped to 250 °C at a rate of 5 °C min^−1^ and held at this temperature for 8 hours. Finally, the sample was allowed to cool naturally to room temperature before collection.

The obtained powder was subjected to acid treatment in 0.05 M H_2_SO_4_ at 80 °C for 12 hours, with the solution first saturated with argon gas. After this treatment, the catalyst was rinsed repeatedly with ultrapure water until the supernatant reached a neutral pH, and the solid was recovered by centrifugation. The catalyst was then dried thoroughly under vacuum.

Finally, the dried powder was annealed again at 900 °C for 2 hours under a 5% H_2_/Ar gas mixture, using the same heating rate of 5 °C min^−1^. After cooling to room temperature, the collected sample was denoted as the PtCo/C-3 catalyst. The PtCo/C-1, PtCo/C-2, and PtCo/C-4 catalysts were prepared following an identical procedure, using 9, 18, and 54 mg of Co(acac)_2_, respectively, while keeping the amounts of Pt(acac)_2_ (80 mg) and the Vulcan XC 72R carbon support (50 mg) constant.

#### 2.1.2 Physical characterization

X-ray diffraction (XRD) analysis was performed using a third-generation Malvern Panalytical Empyrean system, equipped with multicore (iCore/dCore) optics and a Pixcel3D detector operated in one-dimensional scanning mode. Co Kα radiation (*λ* = 1.7890 Å) served as the X-ray source for phase identification. Diffraction patterns were collected over a 2*θ* range of 20° to 110°, with a step size of 0.02° and a scan time of 110 seconds per step. The obtained patterns were analyzed using Malvern Panalytical's Highscore Plus software (version 4.9) and the ICDD PDF-4+ reference database.

X-ray photoelectron spectroscopy (XPS) measurements were conducted using a Kratos Axis Ultra DLD system at the University of Warwick. The samples were mounted on an electrically isolated holder using conductive carbon tape to minimize charging effects. All spectra were recorded at room temperature using a monochromatic Al Kα source (1486.7 eV), with an analysis area of 300 microns × 700 microns, a take-off angle of 90° and a pass energy of 20 eV for high-resolution core-level spectra (resulting in an energy resolution of approximately 0.4 eV). To prevent surface charging of the surface, the sample was flooded with a low-energy electron beam from a charge neutralizer throughout the measurement; this required recalibration of the binding energy scale. Energy calibration was initially performed with a silver standard, with the sp^3^ C–C/C–H component of the C 1s spectrum set to 285.0 eV as the final charge reference. Spectral fitting and quantitative analysis were carried out using CasaXPS, employing Shirley-type backgrounds and Voigt line shapes, with asymmetry parameters where appropriate.

Annular dark-field and bright-field scanning transmission electron microscopy (ADF/BF-STEM) combined with energy-dispersive X-ray (EDX) mapping was performed using a JEOL ARM200CF instrument. This microscope is equipped with dual aberration correction and was operated at an accelerating voltage of 200 kV. EDX analysis was carried out using a windowless Oxford Instruments detector with a sensor area of 100 mm^2^.

Elemental quantification was conducted by inductively coupled plasma optical emission spectrometry (ICP-OES) using a Thermo Scientific iCAP 7400 instrument. Approximately 10 mg of catalyst samples were digested in a mixture of aqua regia and hydrofluoric acid (HF) under microwave heating before analysis.

### 2.2 Electrochemical measurements of catalyst activity

Electrochemical measurements of catalyst activity were performed using a standard three-electrode configuration connected to a Solartron 1287A potentiostat. The catalyst-coated rotating disk electrode (RDE) served as the working electrode, a graphite rod (5 mm in diameter) as the counter electrode, and an Ag/AgCl electrode as the reference. All tests were conducted either in 1.0 M KOH or in 1.0 M KOH containing 0.1 M NH_3_.

A homogeneous catalyst ink was prepared by dispersing 5 mg of the catalyst powder in 950 µL of isopropanol, followed by the addition of 50 µL of 5 wt% Nafion solution and sonication for 30 minutes. A 20 µL aliquot of the ink was drop-cast onto a pre-polished glassy carbon RDE with a geometric area of 0.196 cm^2^, resulting in a total catalyst loading of ∼0.51 mg cm^−2^. Based on the metal content determined by using inductively coupled plasma mass spectrometry (23.86 wt% Pt for the PtCo/C-3 catalyst), the corresponding noble metal loading on RDE was calculated to be 0.122 mg cm^−2^.

Before measurement in 1.0 M KOH, the working electrode was electrochemically activated by performing 30 cyclic voltammetry (CV) cycles from −0.8 to 0.5 V (*vs.* Ag/AgCl) at a scan rate of 50 mV s^−1^ under argon saturation. Subsequent CV characterization was conducted at a scan rate of 10 mV s^−1^ with an electrode rotation rate of 900 rpm. For the ammonia oxidation reaction (AOR) test in the NH_3_-containing electrolyte, linear sweep voltammetry (LSV) was recorded under identical rotation and scan rate conditions without prior activation.

### 2.3 Gas diffusion electrode fabrication and electrochemical testing

The catalyst ink for the gas diffusion electrode (GDE) was prepared by sonicating a mixture of 30 mg catalyst powder, 2 mL isopropanol (IPA), and 150 µL of 5 wt% Nafion solution for 30 minutes. The resulting ink was uniformly coated onto a piece of hydrophilic carbon cloth to achieve a targeting catalyst loading of approximately ∼5.0 mg cm^−2^. Electrochemical performance of the GDE was evaluated in a three-electrode cell using a Pt foil counter electrode and an Ag/AgCl reference electrode. The electrode was first activated in 1.0 M KOH by performing 30 CV cycles between −0.8 and 0.5 V (*vs.* Ag/AgCl) at 100 mV s^−1^. This was followed by 10 additional CV cycles at a slower scan rate of 20 mV s^−1^ until a stable current response was achieved. Data from the final cycle were used for analysis.

### 2.4 Ammonia fuel cell test

In direct ammonia fuel cell (DAFC) measurements, the PtCo/C-3 electrode was used as both the ammonia oxidation reaction (AOR) anode and the oxygen reduction reaction (ORR) cathode. The anode PtCo/C-3 loading was approximately 5.0 mg cm^−2^, corresponding to a Pt loading of ∼1.19 mg cm^−2^. For the cathode, a PtCo/C-3 loading of ∼3.0 mg cm^−2^ was used, giving a Pt loading of ∼0.71 mg cm^−2^. The effective geometric area of the fuel cell was 1 × 1 cm^2^. A concentrated NH_3_ feed of 7 M was selected for the complete DAFC because the reactant-transport conditions in the porous anode are substantially more demanding than those in the half-cell and carbon-paper electrode measurements. During high-current-density operation, NH_3_ is continuously consumed within the catalyst layer, and diffusion resistance through the flow field, porous electrode, and ionomer phase may lead to local fuel depletion and severe concentration polarization. Increasing the bulk NH_3_ concentration enhances the concentration driving force for NH_3_ transport, improves reactant accessibility to the inner active sites, and helps sustain the AOR rate at high current densities. This selection is also consistent with recent device-level DAFC studies in which concentrated NH_3_ feeds, including 7 M and 11 M NH_3_, improved peak power density by alleviating anode mass-transport limitations.^39,40^ Meanwhile, 3 M KOH provides sufficient OH^−^ for the successive dehydrogenation steps of the AOR. Therefore, 7 M NH_3_ was adopted as an appropriate device-level operating concentration for the present PtCo/C-3 cell, rather than being regarded as a universally optimal concentration for all DAFC systems. An anolyte solution of 7 M NH_3_ + 3 M KOH was pumped into the anode at a flow rate of 1 mL min^−1^ under a back pressure of 2 bar. The anolyte consisting of 7 M NH_3_ in 3 M KOH was pumped into the anode flow field at a flow rate of 2 mL min^−1^. Meanwhile, pure O_2_ was passed through a humidifier maintained at 100 °C at a flow rate of 120 mL min^−1^ before entering the cathodic chamber, with the back pressure maintained at 3.0 bar. Temperature regulation of the fuel cell was achieved using cartridge heaters, with real-time monitoring provided by integrated thermocouples. Electrochemical performance was evaluated utilizing a Solartron 1287A potentiostat/galvanostat connected to a Solartron 1260 impedance analyzer, controlled by CorrWare and ZPlot software suites. EIS spectra were acquired in the frequency range from 1 MHz to 0.01 Hz with an amplitude of 10 mV for the AC perturbation. Polarization data were collected at various operating temperatures using a potential sweep rate of 5 mV s^−1^. For benchmarking, a commercially available anion exchange membrane (Fumapem FAA-3-50-CCM3, 50 µm thick) was tested under identical conditions.

## 3 Results and discussion

### 3.1 Catalyst preparation and physical characterization

**Fig. 1:**
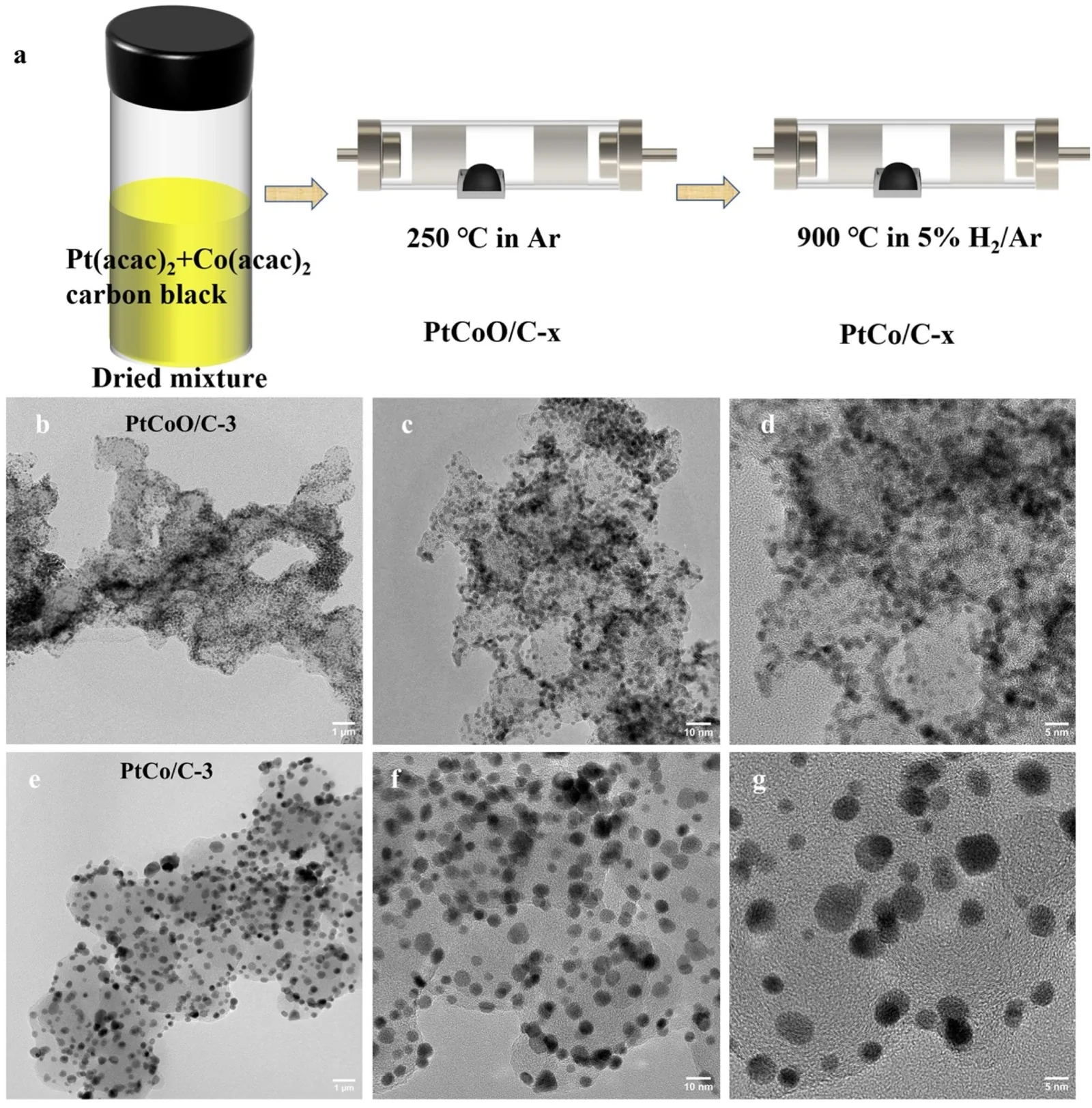
(a) Synthesis process of the PtCo/C catalyst; (b–d) TEM images of PtCoO/C-3 (∼3 nm); (e–g) TEM images of PtCo/C-3 (4–6 nm).

A two-step pyrolysis synthesis process was employed, as illustrated in Fig. 1a. In the first pyrolysis stage, the molecular precursors platinum(ii) acetylacetonate and cobalt(ii) acetylacetonate undergo gradual thermal decomposition in a pure argon atmosphere at 250 °C. This low-temperature, oxygen-free environment selectively reduces the Pt^2+^ ions, leading to the formation of metallic platinum nucleation sites. Concurrently, the cobalt species form amorphous cobalt oxide (CoO) clusters that are uniformly and intimately distributed with Pt across the carbon support surface. This results in a finely mixed yet unalloyed composite PtCoO/C material with an average diameter of approximately 3 nm, in which the distinct metallic and oxide phases remain separate (Fig. 1b–d). In the second critical stage, the atmosphere is switched to a gas mixture containing 5% H_2_ balanced with argon. In this mildly reductive environment, a carefully programmed temperature ramp is applied. This controlled heating facilitates the gradual reduction of the amorphous cobalt oxide species to metallic cobalt atoms. These nascent Co atoms subsequently diffuse into the existing face-centered cubic lattice of the pre-formed Pt nanocrystals. This slow, solid-state interdiffusion process is key to forming homogeneous Pt–Co alloy nanoparticles, thereby achieving the targeted alloying phase.

**Fig. 2:**
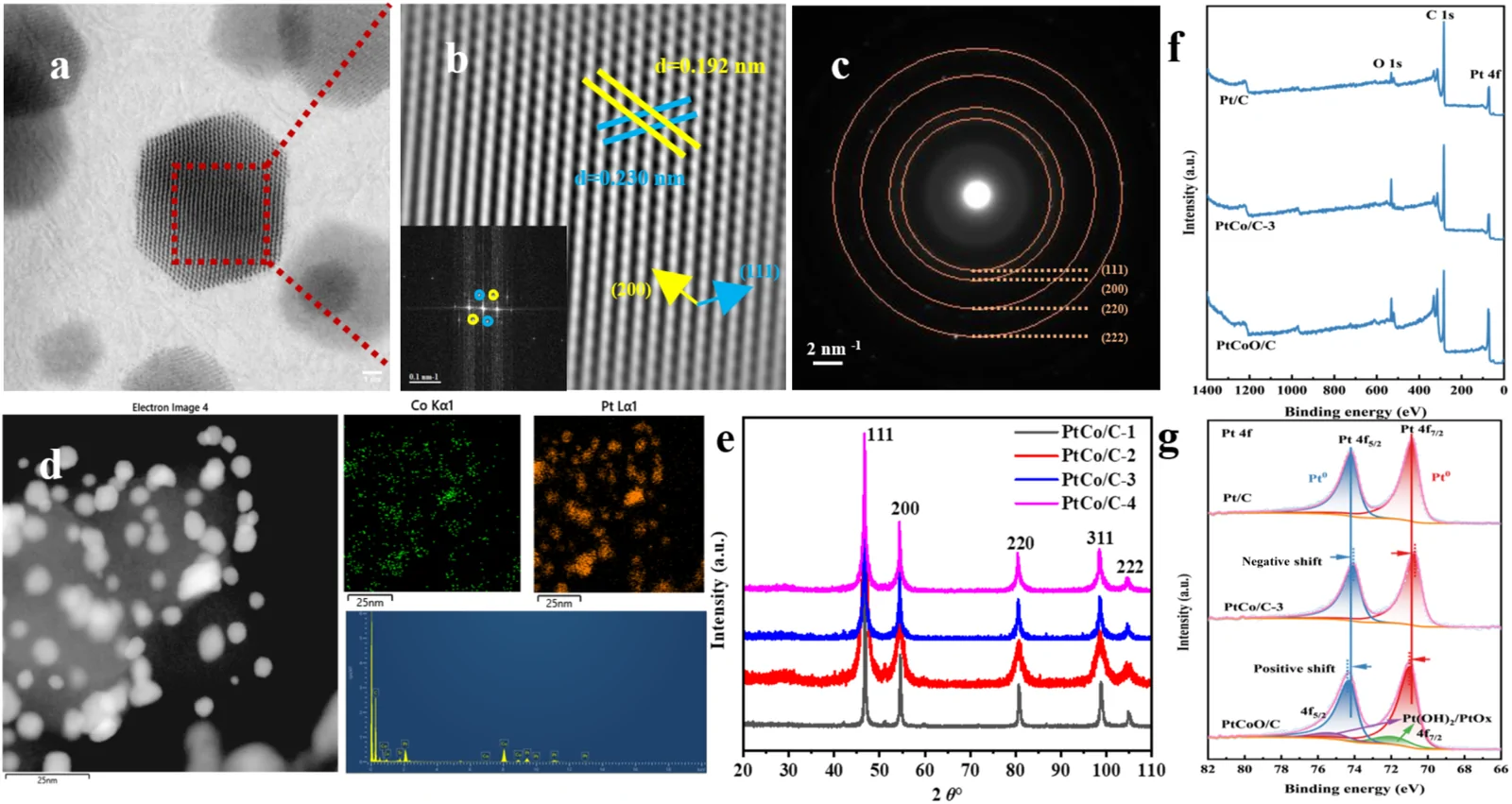
(a and b) HRTEM images; (c) SAED images; (d) EDX-mapping of PtCo/C-3; (e) XRD patterns of PtCo/C-*x* (*x* = 1, 2, 3, 4) and Pt/C; (f) XPS survey spectra and (g) high-resolution Pt 4f spectra of Pt/C, PtCo/C-3, and PtCoO/C.

Morphological analysis *via* transmission electron microscopy (TEM) reveals that finely divided PtCo nanoparticles are homogeneously deposited across the carbon support matrices (Fig. 1e–g). High-resolution TEM (HRTEM) imaging successfully resolves characteristic lattice fringes of the crystal planes of the metallic phases. The corresponding fast Fourier transform (FFT) pattern confirms the crystallinity, showing spots corresponding to the (200) and (111) planes (Fig. 2a and b). Inverse FFT (IFFT) filtering of specific diffraction spots for PtCo/C-3 resolves clear lattice fringes with spacings of 0.192 nm and 0.230 nm, corresponding to the (200) and (111) planes, respectively^41^ (Fig. 2b). The selected-area electron diffraction (SAED) pattern of the PtCo/C-3 catalysts is indexed to the (111), (200), (220), and (222) facets of a face-centered cubic (FCC) structure^35^ (Fig. 2c). EDX elemental mapping (Fig. 2d) was performed to characterize the spatial distribution of Pt and Co in the PtCo/C-3 catalyst. Although the Co signal is relatively weak due to its low bulk content, its distribution pattern matches well with that of Pt, demonstrating that residual Co atoms are uniformly embedded in the Pt matrix without the formation of segregated Co-rich phases. Semi-quantitative EDS analysis (Table S1) provides a surface elemental composition of approximately 6.79 at% Pt and 0.15 at% Co (Pt/Co ≈ 45 : 1) for PtCo/C-3, which is only used for a preliminary evaluation of elemental dispersion uniformity. The precise bulk elemental composition was further determined *via* ICP-OES measurements. The results reveal a total metal loading of 24.03 wt% (23.86 wt% Pt and 0.17 wt% Co), corresponding to an atomic ratio of 97.7 at% Pt and 2.3 at% Co (Pt/Co ≈ 42.4 : 1). Notably, all quantitative compositional data reported in this work are exclusively derived from the ICP-OES results. XRD analysis shows diffraction peaks for pure Pt. Upon Co incorporation, all peaks shift to higher angles, indicating lattice contraction due to the smaller atomic radius of Co (Fig. 2e and S1).^22,35,42^

X-ray photoelectron spectroscopy (XPS) survey spectra were collected to verify the elemental composition and surface chemical states of the as-prepared PtCo/C and PtCoO/C catalysts (Fig. 2f). High-resolution spectra of Pt, C, and O were further analyzed to quantify the relative surface elemental contents and elucidate the chemical state evolution of active Pt species. The corresponding C 1s and O 1s spectra are displayed in Fig. S3, and the detailed O 1s binding energy values of different samples are summarized in Table S3. Notably, no obvious Co 2p characteristic signal was observed for the PtCo/C sample in the XPS survey spectrum, despite the unambiguous presence of Co (*ca.* 2.3 at%) confirmed by ICP-OES bulk composition analysis. This implies that Co species are predominantly located in the subsurface or bulk region, with minimal surface exposure, rendering them undetectable by XPS due to its limited probing depth (approximately 2–5 nm). Meanwhile, no cobalt oxide passivation layer was formed on the outer surface to shield Co signals.

The high-resolution Pt 4f XPS spectra of all samples were deconvolved into two characteristic doublets corresponding to Pt 4f_7/2_ and Pt 4f_5/2_ orbitals (Fig. 2g). For the PtCoO/C-3 precursor, the Pt 4f binding energies were positively shifted to 70.95 eV and 74.35 eV, respectively (Table S4). After calcination and reduction, the Pt 4f binding energies of PtCo/C-3 negatively shifted to 70.77 eV and 74.07 eV, while pure Pt/C exhibited intermediate Pt 4f peak positions at 70.84 eV and 74.20 eV. The slight negative shift of Pt 4f peaks observed for PtCo/C is consistent with the typical electron transfer behavior from electropositive Co to electronegative Pt in Pt–Co alloy systems, as widely reported in previous studies.^22,35,37,38^ Such electronic modulation increases the surface electron density of Pt atoms, which is speculated to weaken the adsorption of electron-accepting nitrogen-containing intermediates (*e.g.*, *N) and improve the anti-poisoning performance of the catalyst during the AOR.

The O 1s spectra provide supplementary evidence for the surface electronic variation (Fig. S3 and Table S3). Compared with pure Pt/C, both PtCo/C and PtCoO/C display slightly negatively shifted O 1s binding energies, indicating increased electron density of surface oxygen-containing species, which is consistent with the electron enrichment of Pt induced by Co doping. Moreover, PtCo/C exhibits a significantly larger O 1s peak area than Pt/C, demonstrating a higher abundance of surface adsorbed oxygen and hydroxyl groups. This phenomenon is reasonably attributed to the electronic modulation effect induced by Co incorporation, which alters the surface adsorption behavior of oxygen-containing species.

The above qualitative speculation is strongly supported by previous mechanistic studies on Pt-alloy AOR catalysts.^22,35^ A large number of *operando* spectroscopic tests and DFT calculations have confirmed that alloying Pt with transition metals (Co, Cu, Zn, *etc.*) can effectively regulate the electronic structure of Pt active sites, weaken Pt–N bonding interactions, promote the desorption of toxic *N and *N_2_ intermediates, and thus enhance the catalytic durability of Pt-based catalysts.^22,35,36,43^ Given the similar electronegativity of Co (1.88) and Cu (1.90), the well-established electronic modulation mechanism of PtCu alloys (intermetallic charge transfer, elevated Pt electron density, downshifted d-band center, and weakened nitrogen intermediate adsorption) is qualitatively applicable to our PtCo/C system.^36^

### 3.2 Electrochemical performance of the ammonia oxidation reaction

**Fig. 3:**
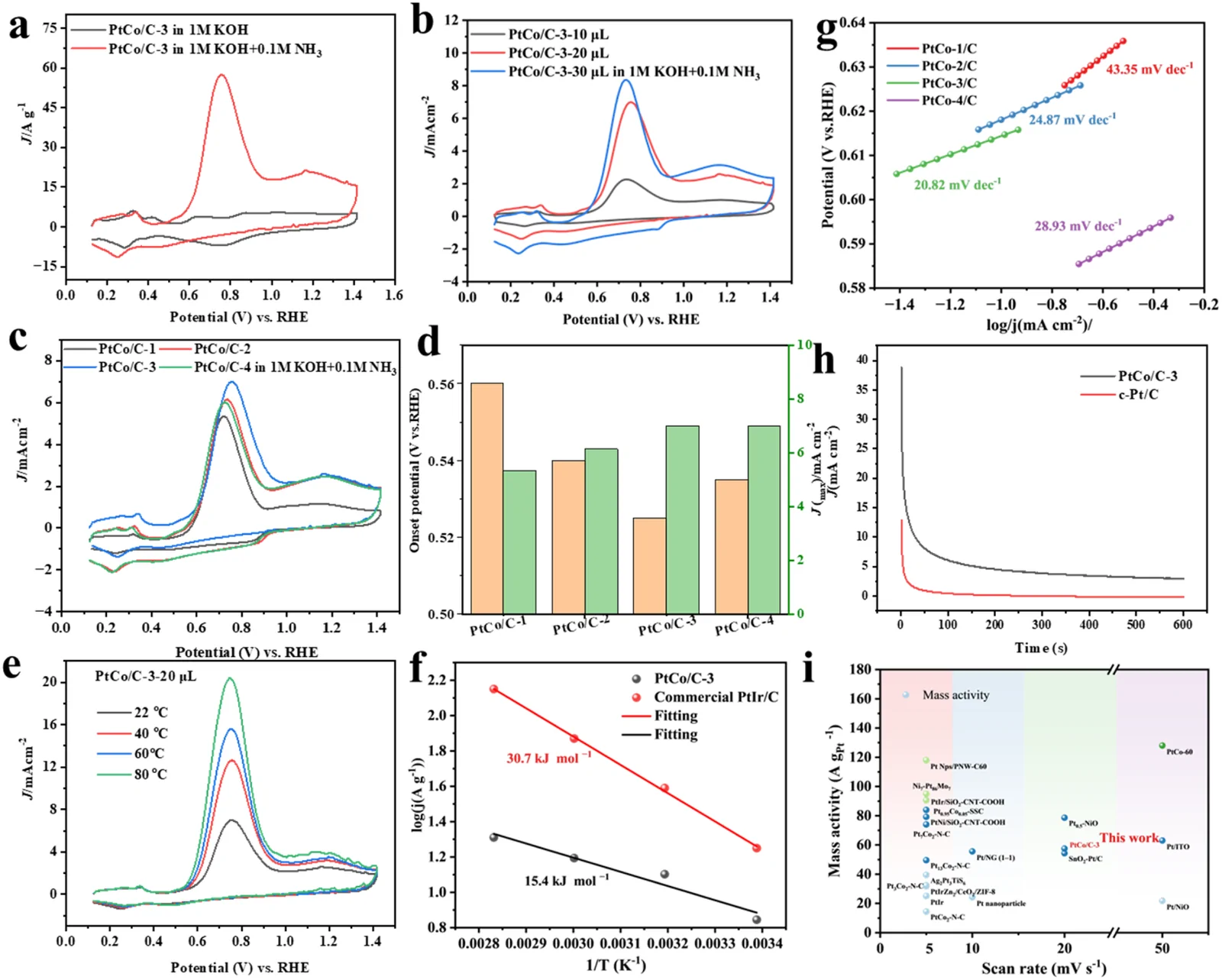
Electrochemical AOR performances. (a) CVs of mass activity of PtCo/C-3 in the presence and absence of NH_3_; (b) CVs of PtCo/C-3 with different loading masses in 1 M KOH + 0.1 M NH_3_; (c) CVs and (d) onset potential and *J*_max_ of PtCo/C-*x*, *x* = 1, 2, 3, 4 in 1 M KOH + 0.1 M NH_3_ at a scan rate of 20 mV s^−1^; (e) CVs of PtCo/C-3 with 20 µL at different temperatures; (f) Arrhenius plots of PtCo/C-3 and commercial PtIr/C; (g) Tafel slope; (h) *J*–*t* plots of PtCo/C-3 at −0.2 V *vs.* Ag/AgCl at 80 °C in 1 M KOH + 0.1 M NH_3_; (i) the comparison of the mass activity of PtCo/C-3 with the reported catalysts for the AOR.

The electrochemical ammonia oxidation reaction (AOR) performance was evaluated using a rotating disk electrode (RDE) in a three-electrode configuration with an electrolyte of 1.0 M KOH containing 0.1 M NH_3_. As shown in Fig. 3a, the addition of NH_3_ to the KOH electrolyte produces a distinct ammonia oxidation peak within the potential range of 0.50–1.0 V *vs.* RHE in the cyclic voltammetry (CV) profiles. Fig. 3b shows that increasing the catalyst loading from 10 to 20 µL doubles the AOR current activity, whereas a further increase to 30 µL results in only a marginal enhancement. This diminished response is likely due to the thicker catalytic layers limiting the accessibility of the innermost active sites, implying the existence of an optimal catalyst loading. To investigate the effect of the Pt/Co atomic ratios on AOR activity, three additional control samples (PtCo/C-1, PtCo/C-2, and PtCo/C-4) were evaluated under identical electrochemical conditions (Fig. 3c). In terms of peak current activity, PtCo/C-3 exhibits the lowest onset potential, indicating its favorable ammonia adsorption properties (Fig. 3d). Both excessive and insufficient Co doping have only a weak modulating effect on the Pt catalyst, resulting in a volcanic relationship between AOR activity and trace Co incorporation in the PtCo/C catalyst. This observation is consistent with previous reports.^35^

The influence of temperature on the catalyst activity was investigated by measuring CV curves at different temperatures (Fig. 3e). As the temperature increased from 22 °C to 80 °C, the activity increased fourfold, demonstrating a strong temperature dependence. The Arrhenius plots of PtCo/C-3 and commercial PtIr/C are shown in Fig. 3f, where log(*J*) exhibits a good linear relationship with 1/*T*, indicating that the reaction mechanism remains unchanged with increasing temperature.^22^ Furthermore, the activation energy of PtCo/C-3 is 15.4 kJ mol^−1^, which is substantially lower than that of commercial PtIr/C (30.7 kJ mol^−1^), suggesting higher intrinsic activity of PtCo/C-3 (Fig. 3f).^22^ The Tafel slopes of the four prepared catalysts follow the order of PtCo/C-3 < PtCo/C-1 < PtCo/C-4 < PtCo/C-2. A smaller Tafel slope corresponds to faster charge-transfer kinetics for the ammonia oxidation reaction, which means a lower overpotential is required to reach a specific current density. Combined with its lowest apparent activation energy and the most negative onset potential among all catalyst samples, the comprehensive analysis of multiple kinetic parameters sufficiently verifies that PtCo/C-3 possesses the optimal AOR kinetic properties.^22^ To evaluate the durability of the PtCo/C-3 catalysts, chronoamperometry experiments were conducted at 0.65 V for 600 s and compared with commercial Pt/C (c-Pt/C) (Fig. 3h). Both catalysts exhibited current density decay over time. Throughout the 600 s test, PtCo/C-3 maintained a current density exceeding 5 mA cm^−2^, significantly higher than that of c-Pt/C, indicating superior AOR durability. In summary, the AOR and chronoamperometry results demonstrate that PtCo/C-3 exhibits superior AOR catalytic activity compared to c-Pt/C. A comparison of mass activities between the as-prepared catalyst and previously reported Pt-based AOR catalysts is presented in Fig. 3i and Table S4. In this work, PtCo/C-3 delivers a competitive mass activity of 57.47 A g_Pt_^−1^ at a scan rate of 20 mV s^−1^, which is comparable to that of multiple Pt-based catalysts synthesized *via* similar conventional routes. It is worth noting that discrepancies in test parameters (*e.g.*, scan rate and NH_3_ concentration) across different literature studies will lead to variations in absolute catalytic activity values. Therefore, this cross-literature comparison is only intended for general performance reference, rather than a rigorous quantitative benchmark evaluation. As shown in Fig. S2, the current density of PtCo/C-3 remains at approximately 80% from the first cycle to the 300th cycle. Specifically, the maximum current of PtCo/C-3 at the 1st cycle is 79.3 mA cm^−2^, which increases to 101.4 mA cm^−2^ at the 100th cycle. By the 300th cycle, the maximum current returns to 79.17 mA cm^−2^, and then declines to 71.0 mA cm^−2^ at the 400th cycle. Overall, the maximum current density of PtCo/C-3 remains at approximately 90% of its initial value from the first cycle to the 400th cycle. TEM images verify that the catalyst nanoparticles after 400 CVs are well preserved without obvious agglomeration or pulverization (Fig. S4a). XRD patterns display characteristic diffraction peaks corresponding to the PtCo alloy phase (Fig. S4b), demonstrating the retention of the alloy crystalline structure after cycling. Quantitative ICP analysis (Table S6) reveals that the proportion of dissolved metal species from the electrode is lower than 3 wt%, indicating the catalyst metals are basically stable on the electrode. Pure Co/C was not included as a control because Co-based materials are well known to be essentially inactive for the AOR in alkaline media, and therefore its contribution to the overall activity is negligible.

### 3.3 Electrochemical performance of PtCo/C on carbon paper

**Fig. 4:**
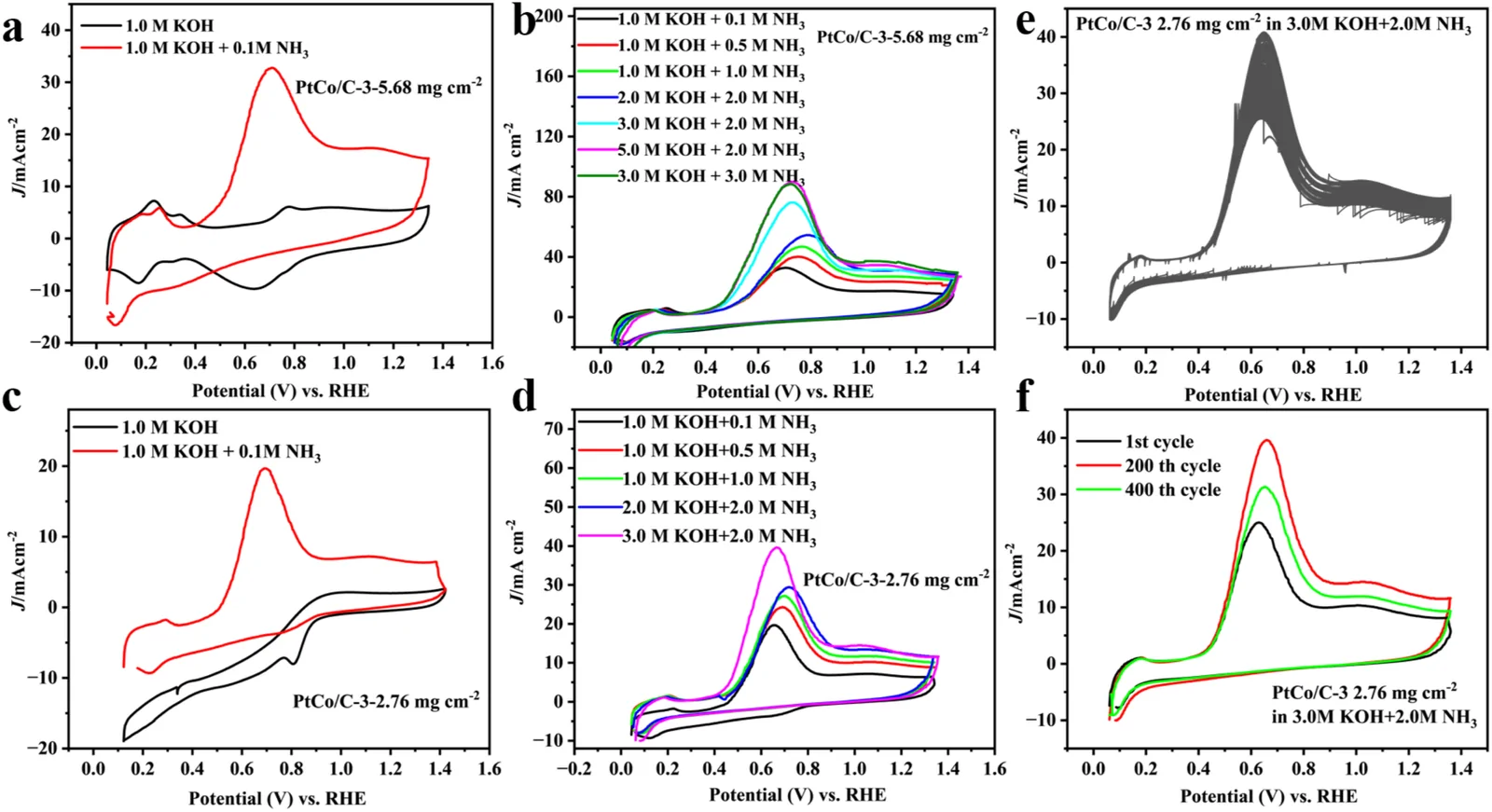
CV performance of PtCo/C-3 on carbon paper. Catalyst loading with 5.68 mg cm^−2^ (a) in 1.0 M KOH and 1.0 M KOH + 0.1 M NH_3_·H_2_O and (b) in different KOH and NH_3_·H_2_O concentrations; catalyst loading with 2.76 mg cm^−2^ (c, e and f) in 1.0 M KOH and 1.0 M KOH + 0.1 M NH_3_·H_2_O, and (d) in different KOH and NH_3_·H_2_O concentrations.

Carbon paper serves as an excellent electrode substrate for DAFCs. To investigate the catalytic activity of PtCo/C toward the AOR on carbon paper, as well as the influence of ammonia concentration and KOH concentration on the performance of carbon paper-based electrodes, electrochemical tests were conducted on the carbon paper electrodes loaded with PtCo/C. The results are shown in Fig. 4. Fig. 4a demonstrates that PtCo/C on carbon paper exhibits good AOR activity. As shown in Fig. 4b, when the KOH concentration is fixed at 1.0 M, and the ammonia concentration increases from 0.1 M to 0.5 M, the maximum AOR current response increases from 32.75 mA cm^−2^ to 54.4 mA cm^−2^, while the corresponding potential increases from 0.78 V to 0.85 V *vs.* RHE. Notably, the onset potential remains largely unaffected by the increase in ammonia concentration. This suggests that the AOR at high ammonia concentration is influenced by concentration diffusion, making the reaction more difficult under such conditions. When the ammonia concentration was fixed at 2.0 M, and the KOH concentration was increased from 1.0 M to 5.0 M, the maximum AOR current response increased from 54.4 mA cm^−2^ to 89.9 mA cm^−2^. Concurrently, the corresponding potential decreased from 0.85 V to 0.78 V *vs.* RHE, and the onset potential (at 10 mA cm^−2^) decreased from 0.6 V to 0.52 V *vs.* RHE. These results indicate that increasing the KOH concentration promotes the kinetics of the AOR.

Comparing Fig. 4a and c, when the catalyst loading increased from 2.76 mg cm^−2^ to 5.68 mg cm^−2^, the maximum current density increased from 19.65 mA cm^−2^ (at a corresponding potential of 0.73 V *vs.* RHE) to 32.75 mA cm^−2^ (at a corresponding potential of 0.78 V *vs.* RHE). However, the potential increased by 50 mV, indicating that while higher catalyst loading enhances AOR reactivity, the associated increase in overpotential is detrimental to the open-circuit voltage of ammonia fuel cells. This observation is consistent with the results obtained on the rotating disc electrode. Furthermore, as shown in Fig. 4b and d, the effects of ammonia and KOH concentration on the AOR at low catalyst loading are essentially consistent with those observed at high loading.

To evaluate the catalyst stability, the CV was performed for 400 cycles under static conditions in a solution of 3.0 M KOH + 2.0 M NH_3_ at a scan rate of 20 mV s^−1^. The results are presented in Fig. 4e and f. The maximum current density at the 1st cycle is 25.3 mA cm^−2^, which increases to 39.6 mA cm^−2^ by the 200th cycle. After 400 cycles, the maximum current density decreased from 39.6 mA cm^−2^ to 25.0 mA cm^−2^, representing a reduction of 21.8%. This decay may be attributed to diffusion limitations, as the test was conducted in a static environment. This issue could be mitigated by flowing the electrolyte during actual DAFC operation. Notably, the accelerated RDE-level stability tests (400 CV cycles and 600 s chronoamperometry) verify the structural robustness and preliminary electrochemical durability of the optimized PtCo/C catalyst. Nevertheless, a comprehensive long-term durability assessment under realistic DAFC single-cell and MEA operating conditions is required for practical application validation, which will be systematically investigated in our future work.

### 3.4 DAFC performance test

**Fig. 5:**
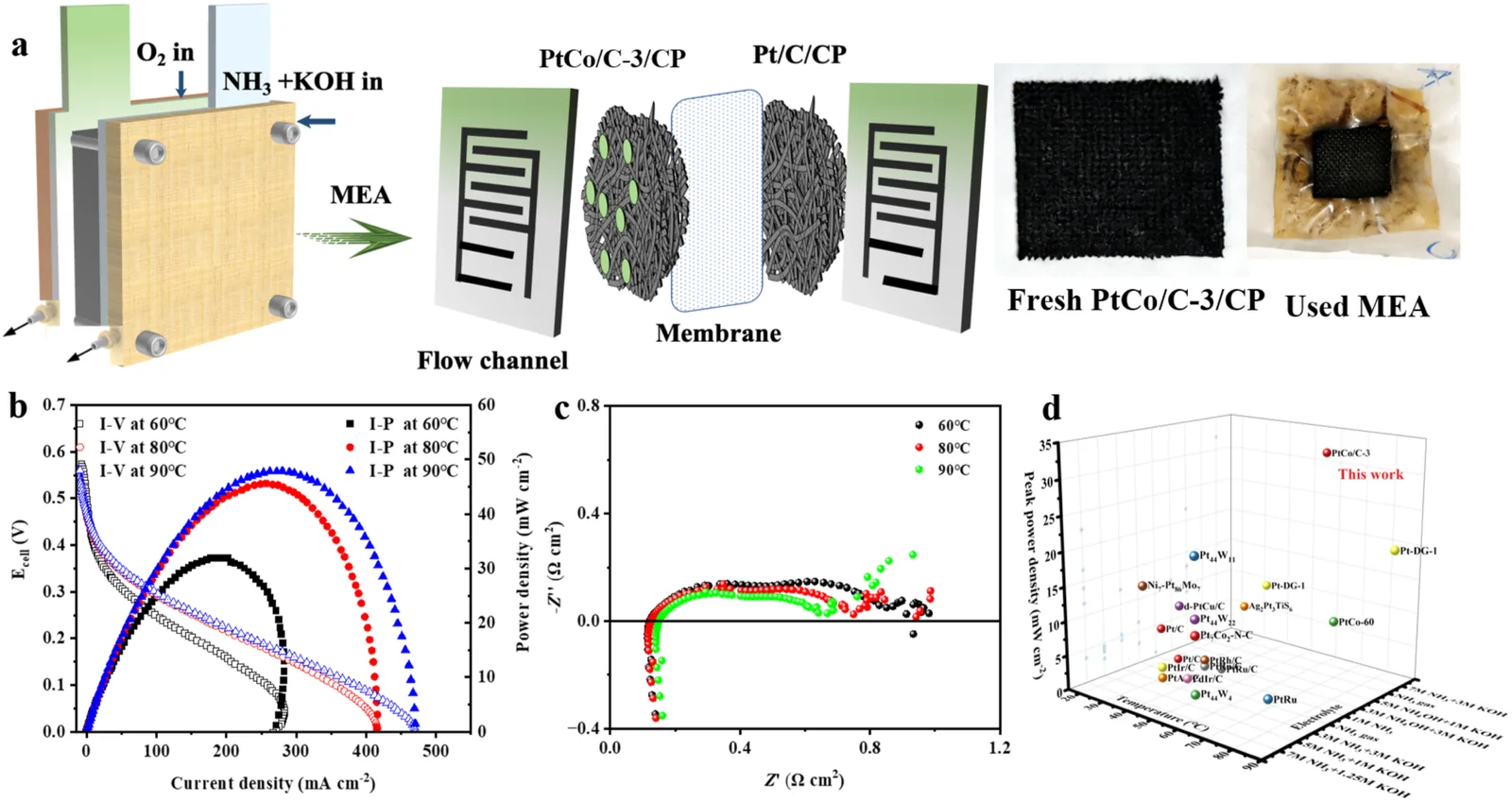
DAFC performance. (a) Schematic diagram of MEA components and the catalyst for a direct ammonia fuel cell; (b) polarization curves and corresponding power density curves of DAFCs with different anodes; (c) cell EIS; (d) comparison of the peak power density of the DAFC with the proposed PtCo/C-3 catalyst as the anode with the reported performance.

The direct ammonia fuel cell consists of a membrane electrode assembly (MEA) and end plate accessories, as illustrated in Fig. 5a. The MEA comprises an anode, a cathode, and an anion exchange membrane (Fig. 5b). Both electrodes are prepared with carbon paper loaded with the PtCo/C-3 catalyst. A photograph of a fresh anode is shown on the left side of Fig. 5c, while the assembled MEA used is shown on the right. As can be seen from Fig. 5b, the PtCo/C-3 catalyst is uniformly coated on the carbon paper electrode. After use, the catalyst remains evenly adhered to the carbon paper.

Fig. 5d shows the performance of the DAFC at different temperatures. At 60 °C, the open-circuit voltage (OCV) is 0.57 V, the limit current density is 270 mA cm^−2^, and the maximum power density reaches 31.9 mW cm^−2^ at 197 mA cm^−2^. When the temperature increases to 70 °C, the limiting current density increases to 415 mA cm^−2^, and the maximum power density reaches 45.6 mW cm^−2^ at 258 mA cm^−2^. Upon a further increase to 90 °C, the OCV is 0.56 V, the limiting current density increases to 472 mA cm^−2^, and the maximum power density reaches 47.9 mW cm^−2^ at 280 mA cm^−2^. These results indicate that increasing the temperature beyond 70 °C does not significantly affect cell performance, which may be related to the performance of the membrane electrode.^44^ A performance comparison of DAFCs assembled with various Pt-based catalysts is presented in Fig. 5f and Table S5. It should be noted that the operating test conditions (*e.g.*, reaction temperature, electrolyte composition, and ammonia feeding mode) vary substantially across different literature studies, making direct performance comparison inappropriate. Under the mild aqueous alkaline operating conditions employed in this work (60 °C, 7 M NH_3_ + 3 M KOH liquid feeding), our PtCo/C-3 catalyst delivers a competitive peak power density of 31.9 mW cm^−2^, which is comparable to most previously reported Pt-based catalysts tested under identical aqueous DAFC conditions. It is worth mentioning that several literature studies have achieved remarkably higher power densities, which are primarily attributed to more harsh operating conditions, such as elevated temperatures or gas-phase ammonia feeding strategies that greatly promote reaction kinetics. While our catalyst and DAFC device show a moderate peak power output compared to high-performance systems, they offer distinct advantages—including a facile synthesis, mild operating conditions, and excellent ammonia tolerance—that make them highly practical and stable for long-term operation in alkaline media.

Notably, the operating potential of the PtCo/C-3 based DAFC in this work falls within the low anodic potential region (≤0.8 V *vs.* RHE), which is the typical operating potential range for the AOR in alkaline fuel cells. Under such low-potential conditions, the AOR proceeds predominantly *via* the dehydrogenation pathway to N_2_, while the formation of toxic NO_2_^−^ and NO_3_^−^ is thermodynamically unfavorable because it requires higher potentials to overcome the kinetic barrier for the oxidation of surface-adsorbed NH_*x*_ intermediates to oxygen-containing species. Venturini *et al.* systematically investigated the potential-dependent selectivity of the AOR on Pt surfaces using online electrochemical mass spectrometry (OLEMS) and ion chromatography (IC), and demonstrated that in the low-potential region of 0.40–0.82 V *vs.* RHE, N_2_ is the major product, while nitrite and nitrate are only detected as solution-phase products of the AOR at high potentials.^39^ Yang *et al.* also provided mechanistic insights into N_2_ formation on Pt in alkaline media, showing that the AOR proceeds *via* dehydrogenation of NH_3_ to NH_2_, followed by NH_2_ coupling to N_2_H_4_ and subsequent dehydrogenation to N_2_.^45^ Therefore, the cell voltage of our DAFC aligns with this selective window, confirming the high selectivity and environmental friendliness of the present symmetric DAFC system.

Our symmetric DAFC uses identical PtCo/C-3 as both the anode and cathode, which simplifies electrode fabrication yet faces two core limitations: mismatched potential windows between the AOR and ORR and poor ammonia tolerance of the cathode catalyst. Ammonia diffuses through the anion exchange membrane driven by concentration gradients. Crossed NH_3_ competes with oxygen for PtCo active sites to retard the ORR and induces parasitic ammonia oxidation on the cathode, lowering the open-circuit voltage and fuel utilization. Alloying Co modulates the Pt electronic structure to deliver decent bifunctional catalytic activity for both the AOR and ORR and strengthen catalyst stability, while an unavoidable trade-off exists: high intrinsic AOR activity accelerates parasitic cathode side reactions once ammonia crossover occurs, and sustained high operating potentials at the cathode lead to more severe decay of PtCo nanoparticles than the anode. We have further deepened the MEA impedance analysis relying on the variable-temperature EIS data in Fig. 5e: membrane resistance increases gradually from 0.12 Ω (60 °C) to 0.125 Ω (70 °C) and finally 0.15 Ω (90 °C), whereas total cell resistance falls from 0.85 Ω to 0.668 Ω as temperature increases, demonstrating that elevated temperature boosts electrode catalytic activity despite worsening membrane ionic conduction. Combined with the DAFC performance curves in Fig. 5d, higher temperature yields larger limiting current density and peak power density, yet performance gains weaken above 70 °C, which we attribute to the increasing membrane resistance offsetting improved electrode kinetics. Since we have not carried out direct characterization of triple-phase boundary evolution in this work, systematic microscopic observation linking impedance variation to TPB structural changes will be implemented in our follow-up research. In subsequent work, we will quantitatively characterize ammonia crossover flux across the membrane under different operating conditions and adjust the alloy composition of PtCo/C to develop bifunctional catalysts with enhanced ammonia tolerance.

## 4 Discussions

This work demonstrates a symmetric direct ammonia fuel cell using a PtCo solid-solution catalyst-coated electrode as both the anode and cathode. This catalyst was successfully synthesized *via* a two-step pyrolysis process. The incorporation of Co atoms induces lattice contraction of Pt and may lead to a negative shift in the Pt 4f binding energy, as suggested by XPS data. The optimal PtCo/C-3 catalyst achieves a peak current density of 79.3 mA cm^−2^ in alkaline medium. It retains approximately 90% of its initial activity after 400 cycles, significantly outperforming pure Pt/C and challenging the conventional notion that high alloying ratios are necessary for enhanced performance. Electrochemical analysis reveals that a high KOH concentration promotes OH^−^-participated dehydrogenation and active site regeneration, whereas an excessively high NH_3_ concentration suppresses the reaction due to diffusion overpotential. This suggests that the AOR is governed by the interplay between surface reaction kinetics and mass transport limitations. In a symmetric direct ammonia fuel cell test, this catalyst delivers a power density of 47.9 mW cm^−2^, validating its feasibility for practical applications. Current challenges for PtCo/C-based DAFCs lie in the performance plateau at elevated temperatures caused by increased membrane resistance, as well as potential surface reconstruction and Co leaching during long-term cycling. To address these issues and advance the practical application of trace-alloying strategies in DAFCs, our future work will focus on multiple targeted research directions. We will optimize PtCo/C alloy composition and construct refined PtCo/CoO_*x*_ interfaces to develop high ammonia-tolerant bifunctional catalysts, adopt low-impedance MEA designs to mitigate temperature-induced performance degradation, and quantitatively characterize membrane ammonia crossover flux under various operating conditions. Furthermore, *in situ* spectroscopic tracking of Co valence evolution and systematic mechanistic analyses combining *in situ* spectroscopy and DFT calculations will be performed to clarify reaction kinetics and resistance differences, fundamentally revealing the structure–performance relationship of the catalysts.

## Author contributions

The work was conceptualized and supervised by S. T. H. Z. performed the catalyst synthesis, catalytic tests, analyzed the data, and wrote the manuscript. P. Z. conducted the catalyst characterization studies, fuel cell tests, and related data processing. Y. S. constructed the membrane electrode assembly for fuel cell tests. Y. H. conducted TEM and HAADF-STEM tests. M. W. performed XPS tests. All the authors discussed the results and commented on the manuscript.

## Conflicts of interest

The authors declare no competing financial interest.

## Supplementary Material

TA-014-D6TA04565K-s001

## Data Availability

Additional datasets or analysis scripts are available from the corresponding author upon reasonable request. Supplementary information (SI): raw electrochemical measurements, material characterization files (XRD, SEM, TEM, and XPS), and supporting spectra. See DOI: https://doi.org/10.1039/d6ta04565k.
