# Supplementary material for: A symmetric direct ammonia fuel cell enabled with poisoning-tolerant PtCo/C electrodes
Source: J Mater Chem A Mater. 2026 Aug 4;14(56):39101–11. doi: 10.1039/d6ta04565k (PMC13599767; doi:10.1039/d6ta04565k)
Supplement: Supplementary file 1 [file TA-014-D6TA04565K-s001.pdf]

Supplementary Information (SI) for Journal of Materials Chemistry A.
This journal is © The Royal Society of Chemistry 2026

Supporting Information

A symmetric direct ammonia fuel cell enabled by poisoning-tolerant PtCo/C electrodes

Huimin Zhang [1, 2], Peimiao Zou[1], Yingjie Song[1], Yisong Han[3], Marc Walker[4], Shanwen Tao[1,5 *]

1 School of Engineering, University of Warwick, Coventry CV4 7AL, UK.

2 School of Civil Engineering and Architecture, East China Jiao Tong University, Nanchang, Jiangxi, 330013, China.

3 Electron Microscopy Research Technology Platform, Department of Physics, University of Warwick, Coventry, UK

4 Photoemission Research Technology Platform, Department of Physics, University of Warwick, Coventry, UK

5 Hartnoll Centre, University of Warwick, Coventry CV4 7AL, UK.

E-mail: s.tao.1@warwick.ac.uk

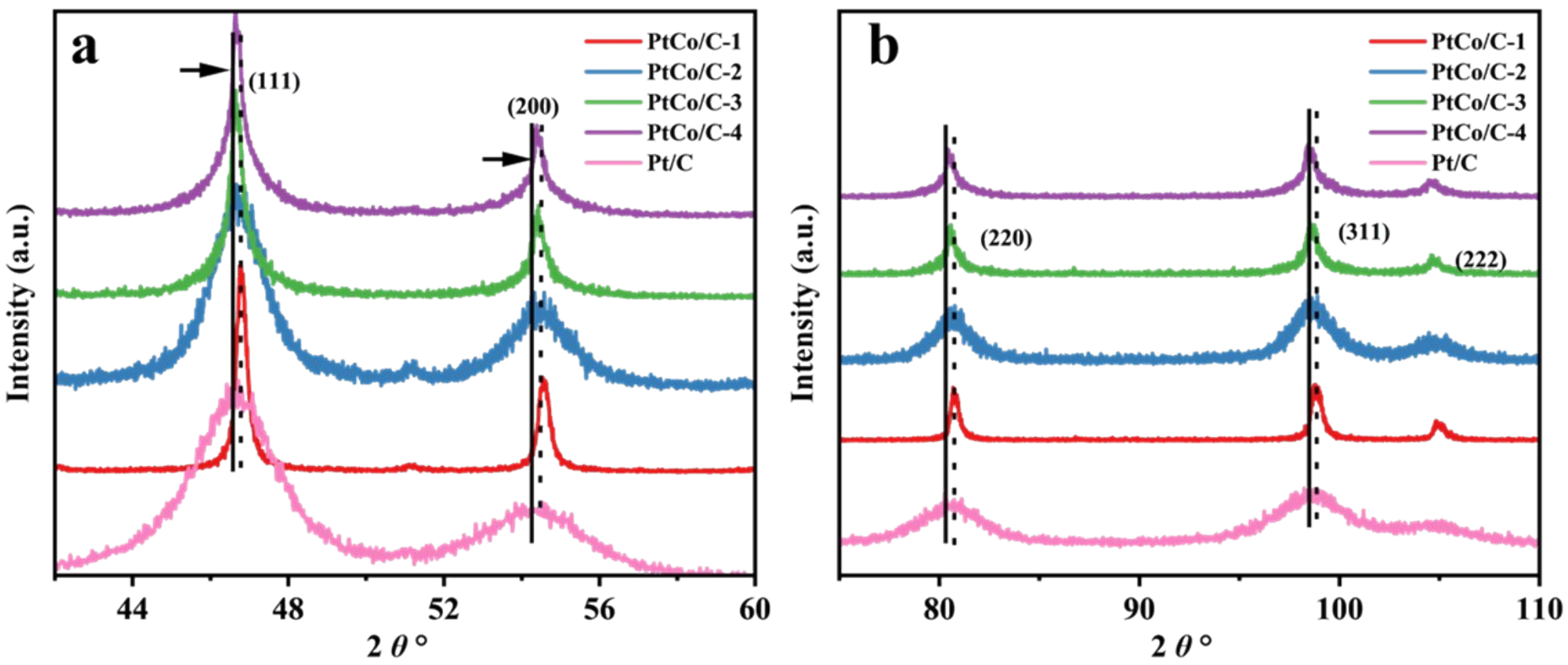

Figure S1 Enlarged view of the Pt diffraction peak for Pt/C and PtCo/C-X catalysts (X = 1, 2, 3, 4).

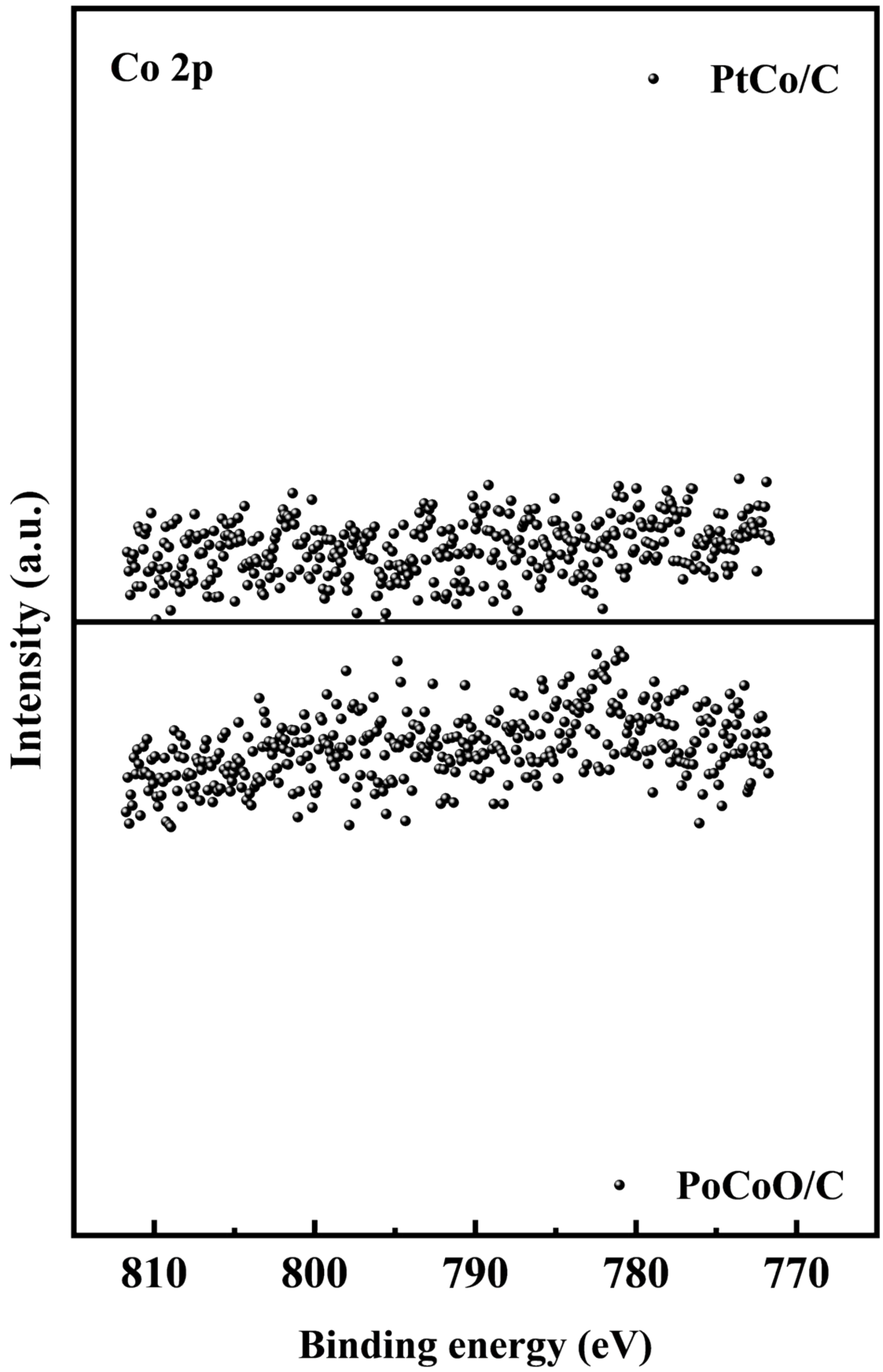

Figure S2 (a) High-resolution Co 2p spectra of PtCo/C-3, and PtCoO/C-3.

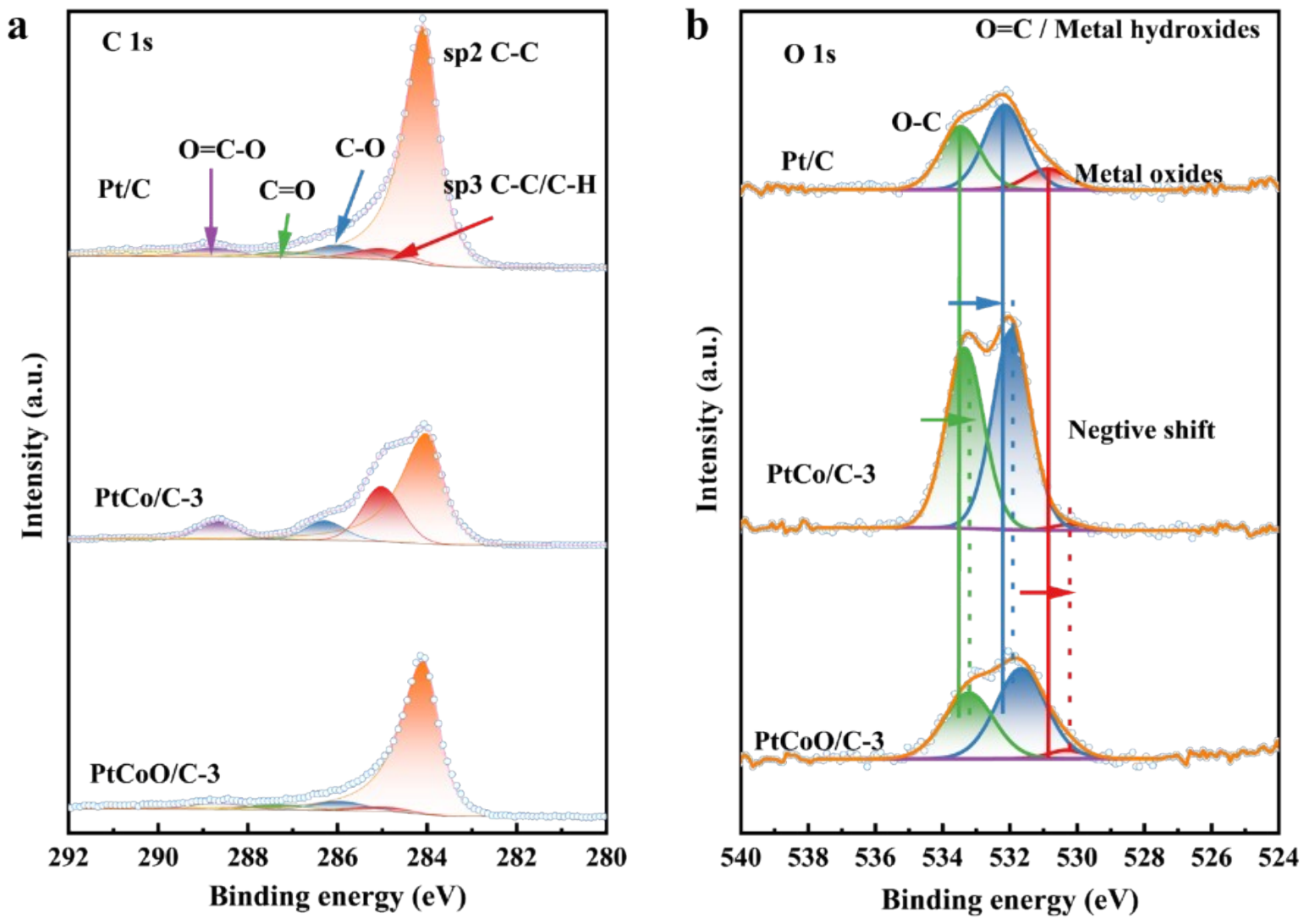

Figure S3 (a) High-resolution C1s spectra and (b) O1s spectra of Pt/C, PtCo/C-3, and PtCoO/C-3.

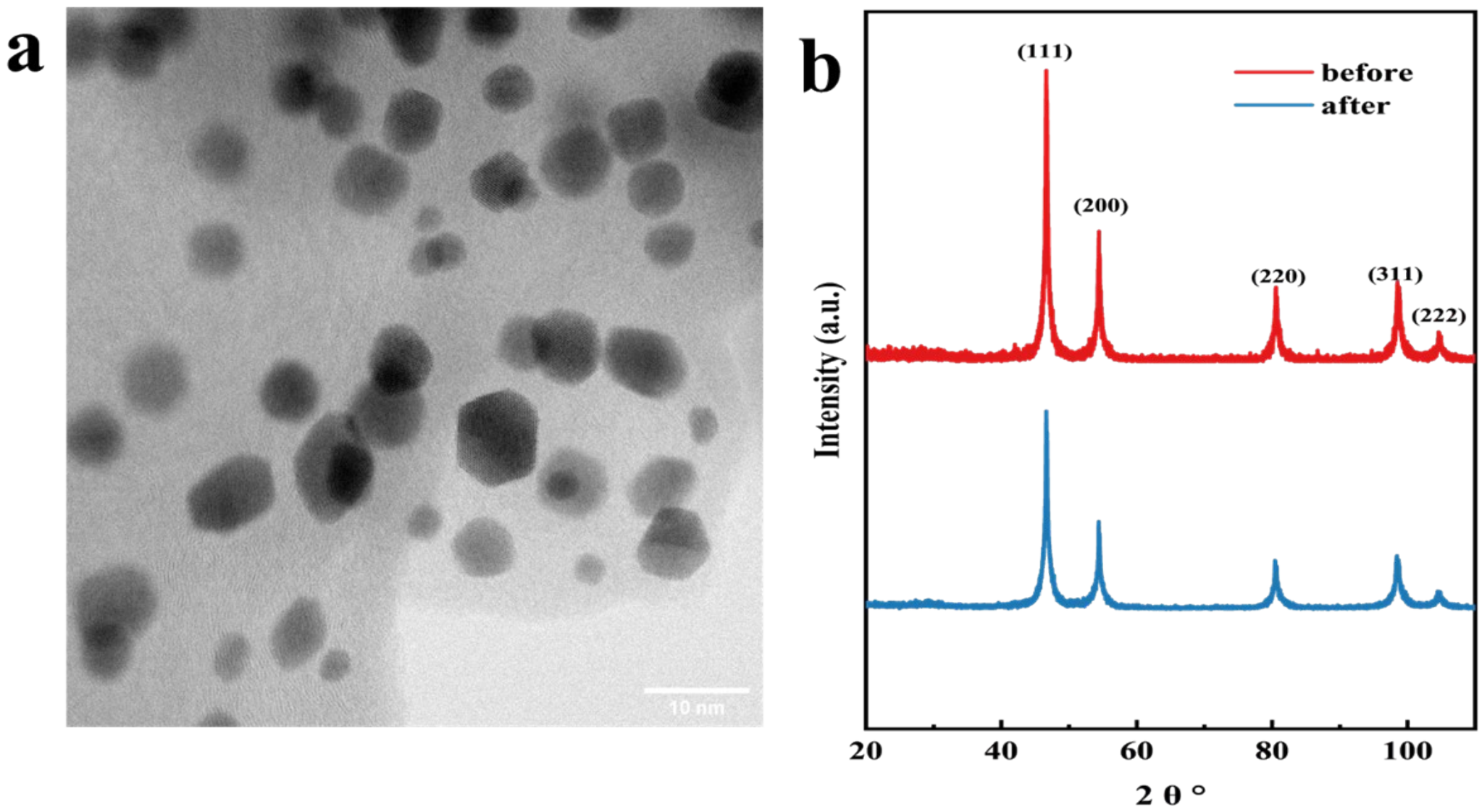

Figure S4 TEM and XRD after 400 CVs of PtCo/C-3.

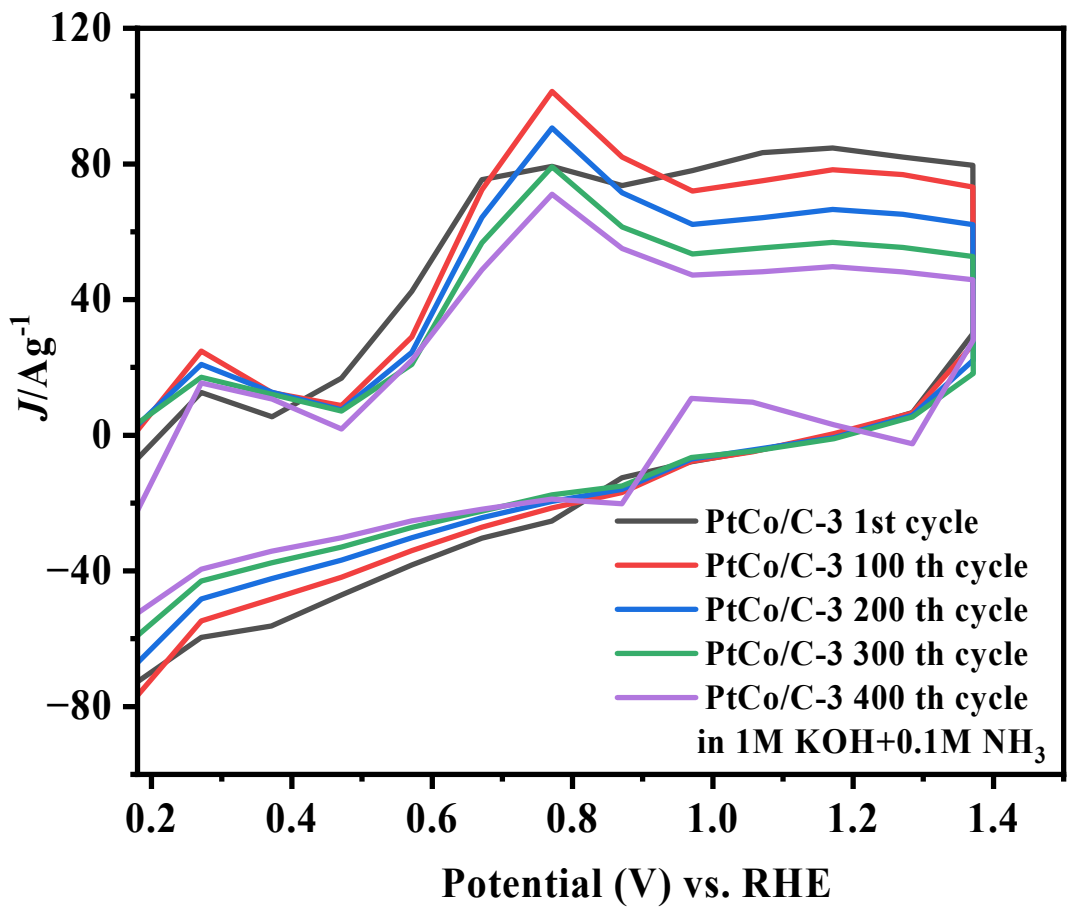

Figure S2 Stability test of PtCo catalyst in 1 M KOH + 0.1 M NH_3 solution.

Table S1. Semi-quantitative EDS elemental composition of the PtCo/C-3 catalyst (average of multiple point-scan analyses).

Element	Average (at.%)	Std. Dev.
C	93.06	3.28
Co	0.15	0.08
Pt	6.79	3.20

Table S2 ICP-OES elemental composition of the PtCo/C-3 catalyst.

Element	Mass fraction (wt. %)	Molar fraction (at. %)	Pt/Co molar ratio
Pt	23.86	97.7	42.4: 1
Co	0.17	2.3	
Total	24.03	100	

Table S3 Binding energy differences observed in high-resolution O 1s spectra for different samples.

Samples	O-C	O=C/MH	M-O
**PtC**	533.5	532.2	530.9
**PtCo/C-3**	533.372	531.972	530.172
**PtCoO/C-3**	533.146	531.646	530.346

Table S4 Binding energy differences observed in high-resolution Pt 4f spectra for different samples.

	**4f5/2(Pt^0)**	**4f7/2(Pt^0)**	**4f5/2($Pt(OH)_2/PtO_x$**	**4f7/2($Pt(OH)_2$/PtOx**
**PtC**	74.2	70.84		
**PtCo/C-3**	74.072	70.772		
**PtCoO/C-3**	74.346	70.946	75.346	71.946

Table S5 Comparison of the AOR performances.

Catalyst	Electrolyte	Scan rate ($mV\ s^{-1}$)	Mass activity ($A\ gPt^{-1}$)	Ref.
PtCo/C-3	1.0 M KOH+0.1 M NH_3	10	57.47	This work
Pt_7Co_2-N-C	1 M KOH+0.1 M NH_3	5	74.04	1
$PtIrZn_2/CeO_2$/ZIF-8	1 M KOH+0.1 M NH_3	5	31.80	2
PtIr	1 M KOH+0.1 M NH_3	5	25.10	3
Pt nanoparticle	1 M KOH+1 M NH_3	10	24.27	4
Pt/NG (1–1)	1 M KOH+0.1 M NH_3	10	55.60	5
SnO_2-Pt/C	1 M KOH+0.1 M NH_3	20	54.29	6
$Pt_{0.5}$-NiO	1M KOH+0.05M $(NH_4)_2SO_4$	20	78.61	7
Pt/NiO	1 M KOH+0.2 M NH_3	50	21.80	8

Pt/ITO	1 M KOH+0.1 M NH_3	50	63.00	[9]
PtCo-60	1 M KOH+1 M NH_4OH	50	128	[10]
Pt-Zn/C nanocubes	0.1 M KOH+0.1 M NH_3	50	405	[11]
Pt-Ir/C nanocubes			535	
Pt-Fe/C nanocubes			352	
Pt-Co/C nanocubes			349	
Pt-Ni/C nanocubes			270	
d-PtCu/C	1.0 M KOH + 0.1 M NH_3		294.4	[12]
$Ag_2Pt_3TiS_6$	0.1 M NH_4OH + 1.0 M KOH	5	39.46	[13]
Pt NPs/PNW-C60	1.0 M KOH + 0.1 M NH_3	5	118	[14]
Pt-DG-1	1.0 M KOH + 0.1 M NH_4OH		1230	[15]
$Pt_{85}Pd_{15}$/rGO	1.0 M KOH + 0.1 M NH_3	5	164.9	[16]
Ni_7-$Pt_{86}Mo_7$	1.0 m KOH + 0.1 m NH_3	5	94.96	[17]
$Pt_{0.95}Co_{0.05}$-SSC	1.0 M KOH + 0.1 M NH_3	5	83.96	[18]

Table S6 Pt and Co metal component on 1cm^{-2} carbon cloth electrode after 400 CV cycles by ICP-OES test

	**initial**	**After 400 CVs**
Pt/mg	1.19	~1.16
Co/μg	8.5	~8.3

Note: The carbon cloth electrode has a catalyst loading of approximately ~5.0 mg cm^{-2}.

Table S7 Comparison of low-temperature DAFC performances.

Anode	Cathode	Electrolyte	Temperature (°C)	Peak power density ($mW\ cm^{-2}$)	Ref.
PtCo/C-3	PtCo/C-3	7M NH_3+3M KOH	60	31.9	This work
Pt_7Co_2-N-C	Pt7Co2-N-C	7 M NH_3+1.25 M KOH	60	11.83	[1]
Pt_7Co_2-N-C	Pt/C	7 M NH3+1.25 M KOH	60	5.97	[1]
$Pt_{44}W_{11}$<br>$Pt_{44}W_4$<br>$Pt_{44}W_{22}$	Pt/C	7 M NH_3+1.25 M KOH	60	22.47<br>3.69<br>14.06	[19]
PtAu/C	Pt/C	5 M NH_3+1 M KOH	40	2.64	[20]
PtIr/C	Pt/C	5 M NH_3+1 M KOH	40	4.17	[21]
PtRh/C	Pt/C	3 M NH_3+3 M KOH	50	5.37	[22]
PdIr/C	Pt/C	5 M NH_3+1 M KOH	50	3.70	[23]
PtRu/C	Pt/C	3 M NH_3+3 M KOH	50	4.5	[22]
PtRu/C	Pt/C	NH_3 gas	50	3.07	[24]
Pt/C	Pt/C	NH_3 gas	25	6.40	[25]
Pt/C	Pt/C	1 M NH_3	25	0.71	[25]
PtRu	Pt	5 M NH_3+1 M KOH	80	4.5	[26]
PtCo-60	20 wt% Pt/C	3 M NH_4OH+3 M KOH	80	11.33	[10]
d-PtCu/C	d-PtCu/C	3 M NH_3+3 M KOH	40	12.2	[12]
Ag_2Pt_3TiS6	Ag_2Pt_3TiS6	5 M NH_4OH+1 M KOH	40	8.71	[13]
Pt-DG-1	Pt/C	1 M KOH+5.0 M NH_4OH	40<br>95	12.8<br>21.8	[15]
Ni_7-$Pt_{86}Mo_7$	Pt/C	1.25 M KOH+7.0 M NH_3	40	16.70	[17]
$Pt_{0.95}Co_{0.05}$-SSC	PtC 40%	NH_3 gas	60	853.75	[18]
$PtIrZn_2/SiO_2$-CNTCOOH		7 M NH_3+1.25 M KOH	95	314	[2]

References

1 F. Fang, Q. Cheng, M. Wang, Y. He, Y. Huan, S. Liu, T. Qian, C. Yan and J. Lu, *ACS Nano*, 2025, **19**, 1260–1270.

2 Y. Li, H. S. Pillai, T. Wang, S. Hwang, Y. Zhao, Z. Qiao, Q. Mu, S. Karakalos, M. Chen, J. Yang, D. Su, H. Xin, Y. Yan and G. Wu, *Energy Environ. Sci.*, 2021, **14**, 1449–1460.
3 Y. Li, X. Li, H. S. Pillai, J. Lattimer, N. Mohd Adli, S. Karakalos, M. Chen, L. Guo, H. Xu, J. Yang, D. Su, H. Xin and G. Wu, *ACS Catal.*, 2020, **10**, 3945–3957.
4 N. Hanada, Y. Kohase, K. Hori, H. Sugime and S. Noda, *Electrochim. Acta*, 2020, **341**, 136027.
5 Y. Zhou, G. Zhang, Z. Gong, X. Shang and F. Yang, *ChemElectroChem*, 2016, **3**, 605–614.
6 T. Okanishi, Y. Katayama, H. Muroyama, T. Matsui and K. Eguchi, *Electrochim. Acta*, 2015, **173**, 364–369.
7 H. Zhang, M. Li, B. Li, Z. Wang and S. Tao, *Chem. Eng. J.*, 2025, **525**, 170434.
8 S. Ntais, A. Serov, N. I. Andersen, A. J. Roy, E. Cossar, A. Allagui, Z. Lu, X. Cui, E. A. Baranova and P. Atanassov, *Electrochim. Acta*, 2016, **222**, 1455–1463.
9 J. Liu, C. Zhong, Y. Yang, Y. T. Wu, A. K. Jiang, Y. D. Deng, Z. Zhang and W. B. Hu, *Int. J. Hydrogen Energy*, 2012, **37**, 8981–8987.
10 C.-E. Hong, D.-H. Park, Y. Gu, S.-H. Park, D.-M. Lim, D.-G. Seo, J.-I. Han and K.-W. Park, *Int. J. Hydrogen Energy*, 2024, **87**, 1367–1376.
11 Y. T. Chan, K. Siddharth and M. Shao, *Nano Res.*, 2020, **13**, 1920–1927.
12 J. Su, J. Wang, Z. Wang, T. K. Kim and J. Wang, *Adv. Funct. Mater.*, 2025, e19518.
13 T. Wu, K. Dhaka, M. Luo, B. Wang, M. Wang, S. Xi, M. Zhang, F. Huang, K. S. Exner and Y. Lum, *Angew Chem Int Ed*, 2025, **64**, e202418691.
14 X. Chen, Z. Ke, X. Wang, H. Jin, Y. Cheng, Y. Xiao, R. Jiang, Y. Da, L. Fan, H. Li, D. Liu, S. Yang and W. Chen, *Angew. Chem. Int. Ed.*, 2025, **64**, e202505180.
15 T. Wu, X. Wang, Q. Yang, B. Wang, R. Yang, S. Chen, L. Chang, S. Wang, F. Huang, Z. Wang and Y. Lum, *Adv. Mater.*, 2025, **37**, e08371.
16 Z. Liu, Y. Li, X. Zhang, S. Rao, J. Li, W. Wang, Z. Sun and J. Yang, *ACS Appl. Mater. Interfaces*, 2022, **14**, 28816–28825.
17 S. Liu, Y. Jiang, M. Wang, Y. Huan, Y. He, Q. Cheng, Y. Cheng, J. Liu, X. Zhou, T. Qian and C. Yan, *Adv. Funct. Mater.*, 2023, **33**, 2306204.
18 Y. He, S. Liu, Q. Cheng, Y. Huan, L. Zhang, W. Shi, W. Shen, F. Zhou, X. Yuan, M. Wang, C. Yan and T. Qian, *J. Am. Chem. Soc.*, 2025, **147**, 28137–28150.
19 Y. Huan, Y. Jiang, M. Wang, X. Zhou, X. Shen, Y. Cao, C. Yan and T. Qian, *Chem. Eng. J.*, 2023, **475**, 146027.
20 J. C. M. Silva, S. G. Da Silva, R. F. B. De Souza, G. S. Buzzo, E. V. Spinacé, A. O. Neto and M. H. M. T. Assumpção, *Appl. Catal., A*, 2015, **490**, 133–138.
21 M. H. M. T. Assumpção, S. G. Da Silva, R. F. B. De Souza, G. S. Buzzo, E. V. Spinacé, A. O. Neto and J. C. M. Silva, *Int. J. Hydrogen Energy*, 2014, **39**, 5148–5152.
22 M. H. M. T. Assumpção, R. M. Piasentin, P. Hammer, R. F. B. De Souza, G. S. Buzzo, M. C. Santos, E. V. Spinacé, A. O. Neto and J. C. M. Silva, *Appl. Catal., B*, 2015, **174–175**, 136–144.
23 M. H. M. T. Assumpção, S. G. Da Silva, R. F. B. De Souza, G. S. Buzzo, E. V. Spinacé, M. C. Santos, A. O. Neto and J. C. M. Silva, *J. Power Sources*, 2014, **268**, 129–136.
24 S. Suzuki, H. Muroyama, T. Matsui and K. Eguchi, *J. Power Sources*, 2012, **208**, 257–262.
25 O. Siddiqui and I. Dincer, *Fuel Cells*, 2018, **18**, 379–388.
26 S. Ishiyama, .
